# Differences between the Influence of Observing One's Own Movements and Those of Others in Patients with Stroke

**DOI:** 10.1155/2019/3083248

**Published:** 2019-07-01

**Authors:** Takeshi Fuchigami, Shu Morioka

**Affiliations:** ^1^Department of Neurorehabilitation, Graduate School of Health Sciences, Kio University, Nara 635-0832, Japan; ^2^Department of Rehabilitation, Kishiwada Rehabilitation Hospital, Kishiwada 596-0827, Japan

## Abstract

We aimed to investigate differences between the influence of observing one's own actions and those of others in patients with stroke with hemiplegia. Thirty-four patients with stroke who had experienced a right or left hemispheric lesion (RHL: n = 17; LHL: n = 17) participated in this study. Participants viewed video clips (0.5× speed) of their own stepping movements (SO) as well as those of others (OO). After viewing the video clips, participants were asked to evaluate the vividness of the mental image of the observed stepping movement using a five-point scale, in accordance with that utilized in the Kinesthetic and Visual Imagery Questionnaire (KVIQ). We also examined changes in imagery and execution times following action observation. When all patients were considered, there were no significant differences between SO and OO conditions. However, in the RHL subgroup, KVIQ kinesthetic subscore and changes in imagery and execution times were greater in the OO condition than in the SO condition. In the LHL subgroup, changes in imagery times were greater in the SO condition than in the OO condition. These findings indicated that viewing the movements of others led to more vivid imagery and alteration in performance in patients with right-sided stroke, when compared to viewing one's own movements. Therefore, the present study suggests that clinicians should consider the side of the damaged hemisphere when implementing action observation therapy for patients with stroke.

## 1. Introduction

Previous studies have suggested that brain activity is similar for motor imagery and action observation [1] and that this similarity is reflected in behavioral outcomes [2]. It has been suggested that the brain activation involved in motor imagery and action observation overlaps with that involved during executed actions [3, 4]. Several previous studies have highlighted the efficacy of neurological rehabilitation interventions involving action observation [5–8]. However, the precise methodology for such interventions remains controversial [9]. Action-observation interventions can be designed using video clips of one's own movements or the movements of others [10]. While some researchers have speculated that viewing self-actions may enhance the vividness of mental imagery, whether this method is more effective than viewing the actions of others remains to be determined.

Previous studies that compared the effect of observing one's own movements versus those of others have utilized mental rotation [11–13] and self-judgment tasks [14, 15]. Such studies reported that viewing one's own body was associated with a higher rate of correct responses than viewing another's body. This effect has been termed “self-advantage” [11]. Furthermore, the effect of self-advantage is decreased in patients with damage to the right hemisphere [12, 14, 15]. The right frontoparietal network has been associated with the domain of self-knowledge, and damage to this network may explain relative decreases in the self-advantage effect [12, 16–19]. Previously, we reported that activity in the right frontoparietal region is high in healthy controls observing self-actions [20], leading us to hypothesize that the effects of observing one's own movements would decrease in patients with damage to the right hemisphere. However, these previous studies only investigated outcomes related to self-knowledge during action observation, and no study has examined changes in performance following action observation.

Previous studies have reported on the changes in the speed of motor imagery and physical execution after action observation [21–23]. These studies used finger movement [22], the arm crank exercise [21], and routine activities executed by the upper extremities [23] as the tasks and presented video clips in which the speed of these movements was irregular. The observations indicated that movement speed became slower after observing the slow video clips, and faster after observing the fast video clips [22, 23]. However, a previous study that measured the motor imagery ability in participants after they listened to music of different tempi suggested that motor imagery was negatively impacted by fast musical tempi relative to the slow musical tempi [24] and, in our experience, action observation of fast movements promotes the effects of counting strategies, i.e., memorizing the pace and rhythm of movements, to be used for later expression [25]. Moreover, in the measurement of imagery times, it is necessary to verify its equivalent in actual execution times [26]. Malouin et al. reported the reliability of measurement of imagery times for stepping movements [27]. Therefore, in the present study, we aimed to investigate the differences in performance after observing one's own actions and those of others, in patients with unilateral cerebral damage, using the observation of slow-speed stepping movements.

## 2. Materials and Methods

### 2.1. Participants and Design

The present study included 34 patients with residual motor impairment on one side of the body resulting from a first cerebrovascular accident. Patients were recruited from the rehabilitation center at Kishiwada Rehabilitation Hospital (Japan). Inclusion criteria were as follows: age between 20 and 85 years and right-handedness. Patients with cerebellar or brainstem lesions, impaired comprehension, moderate to severe receptive aphasia, bodily/visuospatial hemineglect, apraxia, or other neurological conditions (e.g., Parkinson's disease) were excluded. Patient characteristics are presented in Table 1.

All patients provided written informed consent for their participation in the study, which was approved by our hospital's ethics committee (RIN 2016-003).

### 2.2. Experimental Procedures

**Figure 1:**
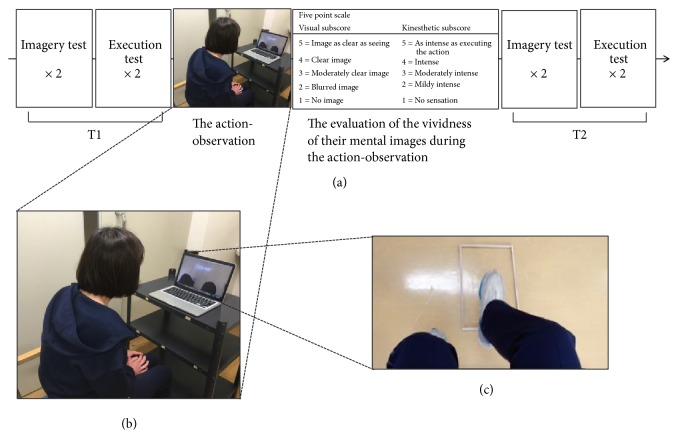
(a) The experimental procedures. Imagery and execution times were assessed before (T1) and after (T2) the action-observation. (b) During the action-observation, the participants were seated in a relaxed position. The video clips were presented at 0.5× speed on a computer screen. (c) The stepping movements presented in the video clips were shown in a first-person perspective. For the self-observation (SO) condition, participants were presented video clips of their own stepping movements. For the other-observation (OO) condition, participants were presented with clips of others performing the stepping movements. The order of the two conditions was randomized.

Imagery and execution times were assessed before (T1) and after (T2) the action-observation (Figure 1(a)). During the task, participants were required to perform stepping movements with the unaffected lower limb while in the sitting position [27, 28]. The stepping movements consisted of placing the unaffected foot forward onto a board and returning it to the floor. The board (41 cm wide × 26 cm long × 2 cm high) was placed transversely, approximately 5 cm in front of the patient's feet. In this task, stepping while seated requires hip flexion to lift the foot from the floor, as well as knee extension/flexion to place the foot on the target and return it to the starting position. This task was chosen because, in contrast to stepping movements performed in the standing position, stepping while seated is considered easier to perform for patients with severe motor and balance impairments. First, the examiner demonstrated the movement once, following which patients performed the movement both mentally and physically a few times before proceeding with formal testing.

During testing, participants sat in a chair with a backrest and were required to imagine and then physically execute a series of five stepping movements at a comfortable speed. The series for imagery and execution were carried out twice each. The imagery test was conducted first to minimize the possibility that the duration of the real movement or counting strategies would influence performance [25]. In the imagery test, participants were instructed to visualize and imagine moving their leg with their eyes closed. The examiner also instructed participants not to execute actual stepping movements or use a counting strategy.

An electronic stopwatch with millisecond-level precision (CASIO Computer Co., Tokyo, Japan) was used to record the durations of imagined and executed movements. During the imagery test, participants used the stopwatch to mark the beginning and end of each trial. During the execution test, the experimenter started and stopped the stopwatch when the participant indicated the start or end of the movement, respectively. Participants were instructed regarding the use of the stopwatch and were allowed to practice prior to formal testing.

For the action-observation, all participants were required to observe video clips of the same stepping movements while seated in a relaxed position (Figure 1(b)). The action-observation included two conditions: self-observation (SO) and other-observation (OO). For the SO condition, participants were presented video clips of their own stepping movements that had been recorded during the execution test. For the OO condition, participants were presented with clips of others performing the stepping movements at the same pace they had utilized in the execution test. In both conditions, video clips were presented at 0.5× speed on a computer screen. The order of the two conditions was randomized. In each video clip, stepping movements were executed five times from the first-person perspective (Figure 1(c)). The video clips were repeated until the number of stepping movements had reached 50. The number was set to 50 for the following reasons: (1) to unify the number of times mental rehearsal was to be performed during action-observation and (2) because the duration of action-observation became approximately 3 minutes as shown in previous studies [21, 22] when the number was set to 50 in a preliminary experiment.

In each trial, participants were required to determine whether the foot shown on the screen was their own or not. During the action-observation, we asked participants to watch the stimuli very carefully and to avoid making bodily movements. In addition, the participants were instructed to imagine with the intention of imitating the stepping movements shown in the video clips.

After the action-observation, participants were asked to evaluate each movement they had experienced in terms of the vividness of their mental images using a five-point scale [29], in accordance with that utilized in the Kinesthetic and Visual Imagery Questionnaire (KVIQ) [30]. The KVIQ assesses both visual and kinesthetic motor imagery and it uses a five-point scale to rate the clarity of the image (visual motor imagery) and the intensity of the sensations (kinesthetic motor imagery); a score of 5 corresponds to the highest level of imagery and a score of 1 to the lowest.

### 2.3. Data Analysis

The mean duration of the two series of five movement repetitions was averaged for each condition (SO and OO) and each test (T1 and T2). To confirm the validity of time measurements [26], the relationship between imagery and execution times was calculated for T1 and T2 using Pearson correlation coefficients. In addition, to determine the influence of each condition, 2×2 repeated-measures analyses of variance (ANOVAs) were performed for condition (SO, OO) and time point (T1, T2), followed by* post hoc* Bonferroni tests for imagery and execution times.

We distributed participants to right and left hemispheric lesion groups (RHL and LHL). We assessed the homogeneity of two subgroups for age and imagery/execution time ratio using unpaired t-test and sex and etiology using chi-square test. The amount of change in imagery and execution times following action observation were calculated for patients with RHLs and LHLs as follows: T2 times-T1 times. The formula describes the degree of influence by action observation, and the numerical value shows that influence by the observation is strong if large. The 2×2 repeated-measures ANOVAs were performed for condition (SO, OO) and subgroup (RHL, LHL), followed by* post hoc* Bonferroni tests for the amount of change in imagery and execution times, KVIQ visual subscore, and kinesthetic subscore.

The level of statistical significance was set at p<0.05, and all statistical tests were performed using SPSS for Mac (IBM Co., Chicago, USA).

## 3. Results

All participants successfully determined whether the foot observed in the video clips was their own or another's.

**Figure 2:**
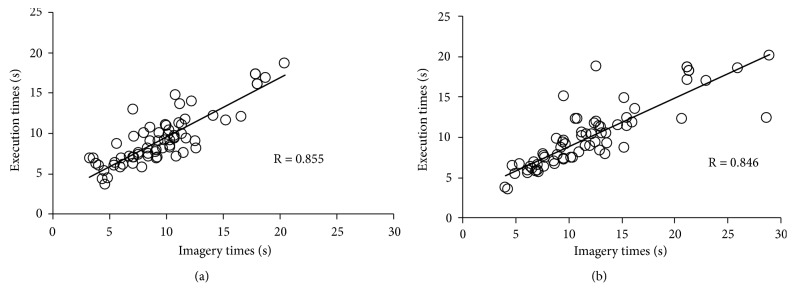
(a) Relationship between imagery and execution times before action observation. (b) Relationship between imagery and execution times after action observation. There was a significant correlation between imagery and execution times both before and after action observation.

We observed a significant correlation between imagery and execution times at both T1 (R=0.855, P<0.001) (Figure 2(a)) and T2 (R=0.846, P<0.001) (Figure 2(b)).

The 2×2 ANOVA revealed no significant interaction for imagery and execution times (imagery: F(1,66)=0.530, P=0.469, execution: F(1,66)=1.343, P=0.251).* Post hoc* tests demonstrated that both imagery and execution times at T2 were significantly longer than those at T1 in the SO and OO conditions (imagery: SO: F(1,33)=21.157, P<0.001, OO: F(1,33)=16.932, P<0.001, execution: SO: F(1,33)=10.679, P=0.003, OO: F(1,33)=16.641, and P<0.001). There were no significant differences between the two conditions for imagery and execution times.

**Figure 3:**
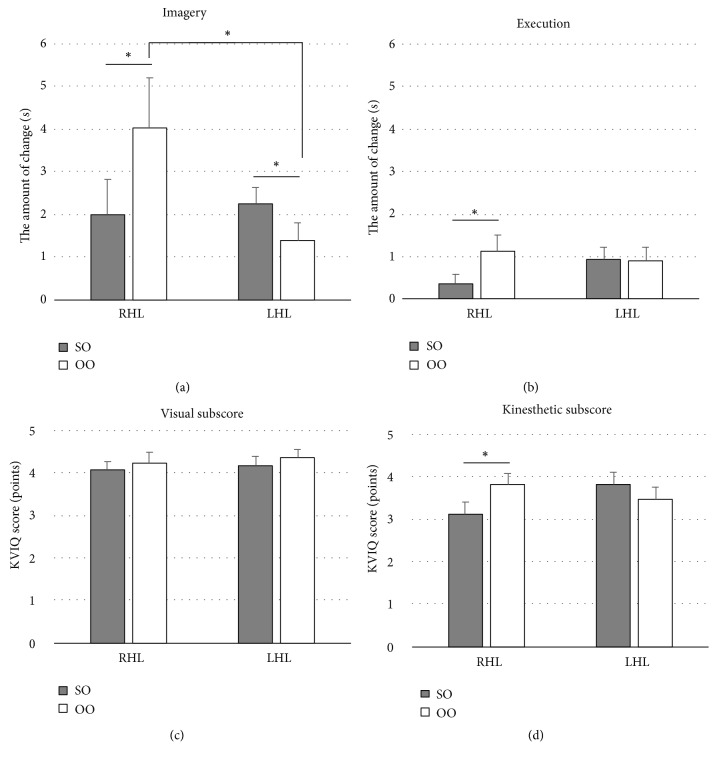
(a) The amount of change in imagery times. In the RHL subgroup, the amount of change in imagery times was significantly larger in the other-observation (OO) condition than in the self-observation (SO) condition. In the LHL subgroup, the amount of change in imagery times was significantly larger in the SO condition than in the OO condition. In the comparison between subgroups, the amount of change in imagery in the OO condition was significantly larger in the RHL subgroup than in the LHL subgroup. The error bars represent the standard error. *∗*Significant difference (P<0.05). (b) The amount of change in execution times. In the RHL subgroup, the amount of change in execution times was significantly larger in the OO condition than in the SO condition. The error bars represent the standard error. *∗*Significant difference (P<0.05). (c) The kinesthetic and visual imagery questionnaire (KVIQ) visual subscore. The error bars represent the standard error. (d) The KVIQ kinesthetic subscore. In the RHL subgroup, the KVIQ kinesthetic subscore was significantly larger in the OO condition than in the SO condition. The error bars represent the standard error. *∗*Significant difference (P<0.05).

The distribution of the participants by age, sex, etiology, and imagery/execution time ratio did not significantly differ between the RHL and the LHL subgroups. The 2×2 ANOVA revealed significant interaction for the amount of change in imagery and execution times and for KVIQ kinesthetic subscore (imagery: F(1,32=9.372, P=0.004, execution: F(1,32)=4.519, P=0.041, KVIQ kinesthetic: F(1,32)=7.758, and P=0.009), although there was no significant interaction in the KVIQ visual subscore.* Post hoc* tests showed that the amounts of change in imagery and execution times and for KVIQ kinesthetic subscore were significantly larger in the OO condition than in the SO condition in the RHL subgroup (imagery: F(1,16)=5.436, P=0.033, execution: F(1,16)=6.210, P=0.024, KVIQ kinesthetic: F(1,16)=4.923, and P=0.041)(Figure 3), although there was no significant difference in the KVIQ visual subscore. In the LHL subgroup, the amount of change in imagery times was significantly larger in the SO condition than in the OO condition (F(1,16)=5.544, P=0.032), although there was no significant difference in the amount of change in execution and KVIQ visual and kinesthetic subscores. In the comparison between subgroups, the amount of change in imagery in the OO condition was significantly larger in the RHL subgroup than in the LHL subgroup (F(1,32)=4.355, P=0.045). There were no other significant differences between the subgroups.

## 4. Discussion

In the present study, we aimed to investigate differences in performance after observing one's own actions and those of others in patients with stroke. When all patients were considered, we observed no significant differences between the SO and the OO conditions. However, in the subgroup analysis, the KVIQ kinesthetic subscore and the amount of change in imagery and execution times were larger in the OO condition than in the SO condition in the RHL subgroup, and the amount of change in imagery times was larger in the SO condition than in the OO condition in the LHL subgroup. These findings indicate that clinicians and therapists should take into account the side of hemispheric damage when considering action-observation therapy for patients with stroke and that videos depicting the actions of others may improve the vividness of imagery and performance in patients with right hemispheric stroke, relative to that with observation of self-actions.

It is necessary to clarify the correlation between imagery and execution times to verify the validity of the temporal equivalence [26]. Our results indicated that imagery and execution times were significantly correlated both before and after the action observation task, highlighting the validity of imagery time measurements in the present study. In the whole analysis, both imagery and execution times significantly increased following observation of the slow stepping movements. The present study is the first to demonstrate that, in patients with stroke, changes in speed lead to immediate alterations in imagery and execution times, extending previous findings observed in healthy participants [21, 22, 24].

For the LHL subgroup, the amount of change in imagery times was larger in the SO condition than in the OO condition. Some studies with healthy and left hemisphere lesion participants have reported self-advantage when observing one's own body versus those of others [11–15] and that the domain responsible for this effect has been considered to be the right frontoparietal regions [12, 16–19]. The right frontoparietal regions have been associated with representations of one's own body [31, 32]. Previously, we reported that these regions are activated during observation of one's own movements [20] and that right hemispheric activation predominates during the observation of self-actions [19, 20]. Therefore, our result pertaining to the LHL subgroup was attributable to self-advantage and is in agreement with previous findings [12, 14].

For the RHL subgroup, the KVIQ kinesthetic subscore and the amount of change in imagery and execution times were larger in the OO condition than in the SO condition. Human brain lesion studies have revealed that damage to the right frontal and parietal cortical regions impairs the perception of one's own body [33] and decreases the self-advantage effect [12, 14, 15]. When we look at our results of the KVIQ kinesthetic subscore (Figure 3(d)) and the amount of change in execution times (Figure 3(b)), our results suggest that “self-disadvantage” [34] occurred due to damage to the right hemisphere in the RHL subgroup. However, there was no difference between the RHL and the LHL subgroups in any SO condition. Moreover, in the OO condition, the amount of change in imagery times was significantly larger in the RHL subgroup than in the LHL subgroup. Thus, it is considered that our results pertaining to the RHL subgroup are attributable to factors other than self-disadvantage; that is, observing others exerted a greater influence in the RHL subgroup.

In the previous study with healthy participants, we showed that the left inferior parietal lobule was activated during observing the movements of others [20]. This region is specialized in associating the neural signal between internal body and external object information [35], and our findings suggested that the body representation of others was converted into own-body representation. Liew et al. reported that the brain activity during action-observation (observing the movement of others) was lateralized towards the left motor hemisphere in patients with stroke [36]. Moreover, one previous study demonstrated that, in motor inhibition, right frontal areas were activated by no-go trials in healthy subjects [37]. In the patients with stroke, hyperactivity of the residual hemisphere occurs because of disintegration of interhemispheric inhibition [38]. In case of right hemisphere lesions, this frontoparietal network becomes disturbed and impulsive responses occur [39]. Hence, patients with RHL might be excessively affected by the movement of others as opposed to their own.

In the LHL subgroup, observation of one's own movements influenced subsequent imagery times, but not execution times. A previous study using a mental rotation task showed that because of laterality judgments on viewing the palm that required more complex sensorimotor computations than judgments on back-displayed hands, the effects of self-advantage were high and suggested that the high sensorimotor load would increase the self-advantage [13]. The video clips that were presented in this study were simple step exercises with only the lower limbs being displayed on the monitor. If video clips that required more complicated sensory integration (i.e., a whole body is displayed on the monitor or the movements were of activities of daily living) were shown, an effect might have been detected. Additionally, in the present study, the execution times were measured after the measurement of imagery times. The effect of self-advantage could not be maintained during this time, and the effect that was found in imagery times might have disappeared.

The results of the KVIQ visual subscore showed no significant differences between the RHL and the LHL subgroups and between the SO and the OO conditions. During action-observation in the present study, we instructed participants to imagine with the intention of imitating the movement. Action-observation with this instruction led to motor system, including frontal cortex, activation [40]. During kinesthetic imagery, motor systems such as those in the frontal and parietal areas are activated [41], whereas visual areas are activated during visual imagery [42]. Thus, a significant difference was found only in the KVIQ kinesthetic subscore. Moreover, in action-observation, it was easier for participants to elicit visual images than kinesthetic images because they watched movements. The results of the KVIQ visual subscore were influenced by a ceiling effect.

This study has several limitations. First, our results cannot be applied to action-observation therapy, as we evaluated performance on the nonparalyzed side to exclude the influence of motor function. However, in accordance with our findings, one previous study reported that action-observation therapy involving movement of others was more effective in patients with right hemispheric damage than in those with left hemispheric damage [43]. Nonetheless, further studies are required to investigate the effects of action observation on performance for the paralyzed side. Second, we did not analyze our results according to lesion domain; instead we divided groups based on the side of the lesion. Damaged regions among participants of the RHL subgroup included the subcortical areas, middle cerebral artery territory, corona radiata, thalamus, frontal cortex, and putamen. Although similar findings have been reported in previous studies [14, 15], these regions are not considered part of the frontoparietal domain involved in the representation of one's own body and in impulsive responses. Future studies should utilize imaging analyses to more fully elucidate the neurological mechanisms underlying the effect in patients with RHL.

## 5. Conclusions

We investigated the influence of observing one's own actions versus those of others in patients with unilateral stroke. In the RHL subgroup, observing the movements of others led to increases in the KVIQ kinesthetic subscore and the amount of change in imagery and execution times compared with the values for the observation of self-actions. Therefore, clinicians and therapists should take into account the side of hemispheric damage when considering action-observation therapy for patients with stroke. Ultimately, our findings suggest that patients with right hemispheric lesions can derive greater benefit from viewing the movements of others than from viewing their own. However, further studies are needed to clarify the mechanism of this effect.

## Figures and Tables

**Table 1:** Participant characteristics.

RHL subgroup (n = 17)						LHL subgroup (n = 17)					
	Age	Sex	Etiology	Lesion location	I/E		Age	Sex	Etiology	Lesion location	I/E
R1	77	M	hemorrhage	subcortical	1.15	L1	65	F	hemorrhage	putamen	0.47
R2	75	M	infarction	MCA territory	1.28	L2	70	M	hemorrhage	thalamus	0.94
R3	83	F	infarction	corona radiata	1.11	L3	85	M	infarction	corona radiata	0.94
R4	76	M	infarction	corona radiata	1.03	L4	79	F	infarction	putamen	1.38
R5	79	M	infarction	corona radiata	0.67	L5	79	F	infarction	temporooccipital cortex	1.54
R6	60	M	hemorrhage	thalamus	0.99	L6	65	M	infarction	corona radiata	1.29
R7	59	M	infarction	frontal cortex	1.32	L7	68	M	infarction	corona radiata	1.34
R8	51	F	hemorrhage	putamen	1.16	L8	63	F	infarction	parietotemporal cortex	1.27
R9	79	M	hemorrhage	putamen	1.12	L9	83	M	infarction	corona radiata	1.20
R10	77	M	hemorrhage	putamen	0.89	L10	57	F	infarction	corona radiata	0.91
R11	73	F	hemorrhage	thalamus	0.95	L11	65	M	infarction	corona radiata	1.19
R12	82	M	infarction	corona radiata	1.13	L12	75	M	infarction	occipital lobe	0.85
R13	45	M	hemorrhage	thalamus	0.92	L13	79	M	hemorrhage	putamen	1.04
R14	68	M	hemorrhage	frontal cortex	1.54	L14	56	F	hemorrhage	thalamus	1.03
R15	47	M	hemorrhage	putamen	1.08	L15	44	M	hemorrhage	putamen	1.03
R16	69	M	infarction	corona radiata	1.11	L16	73	F	infarction	corona radiata	1.13
R17	54	M	infarction	frontal cortex	1.26	L17	61	F	hemorrhage	putamen	1.02

Mean	67.9				1.10	Mean	68.6				1.09
SD	12.7				0.20	SD	10.9				0.25

RHL: right hemispheric lesions; LHL: left hemispheric lesions; I/E: imagery/execution time ratio of stepping movements at first measurement; M: men; F: female; MCA: middle cerebral artery.

## Data Availability

The data used to support the findings of this study are available from the corresponding author upon request.
